# Surgical Strategy for Huge Right Coronary Artery Aneurysm Combined with Left Ventricular Fistula

**DOI:** 10.1155/2021/8438640

**Published:** 2021-10-07

**Authors:** Takafumi Terada, Yoshimori Araki, Akihiro Kobayashi, Osamu Kawaguchi

**Affiliations:** Department of Cardiac Surgery, JA Aichi Koseiren Toyota Kosei Hospital, Aichi, Japan

## Abstract

Coronary artery aneurysms combined with left ventricular fistulas are rare; coronary revascularization strategy after coronary artery aneurysm resection is complex in such cases. We report the surgical repair of a giant right coronary artery aneurysm with a fistula in the left ventricle in a 79-year-old woman diagnosed with an aneurysm 50 mm in diameter. Surgical repair included resection of the coronary artery aneurysm, coronary artery bypass grafting to the posterior descending artery, and isolation of reconstructed right coronary circulation from the fistula. The postoperative course was uneventful; postoperative coronary angiography revealed a patent bypass graft unconnected to the left ventricle.

## 1. Introduction

Coronary artery aneurysm (CAA), defined as local dilatation of the coronary artery more than 150% of the referred coronary artery, is a rare condition detected in 0.3% to 4.9% of patients who undergo coronary angiography [1, 2].

Coronary artery fistula (CAF), defined as an abnormal direct connection between the coronary artery and cardiac chamber or other great vessels, is also a rare anomaly, with an estimated incidence of 0.002% in the normal population [3, 4].

The coexistence of CAA and CAF to the left ventricle (LV) is extremely rare. In this case, we considered CAA as an indicator of operative repair since its diameter was >50 mm. However, the surgical strategy of coronary reconstruction after aneurysm resection is complicated if closure of the fistula to the LV is impossible.

## 2. Case Report

**Figure 1:**
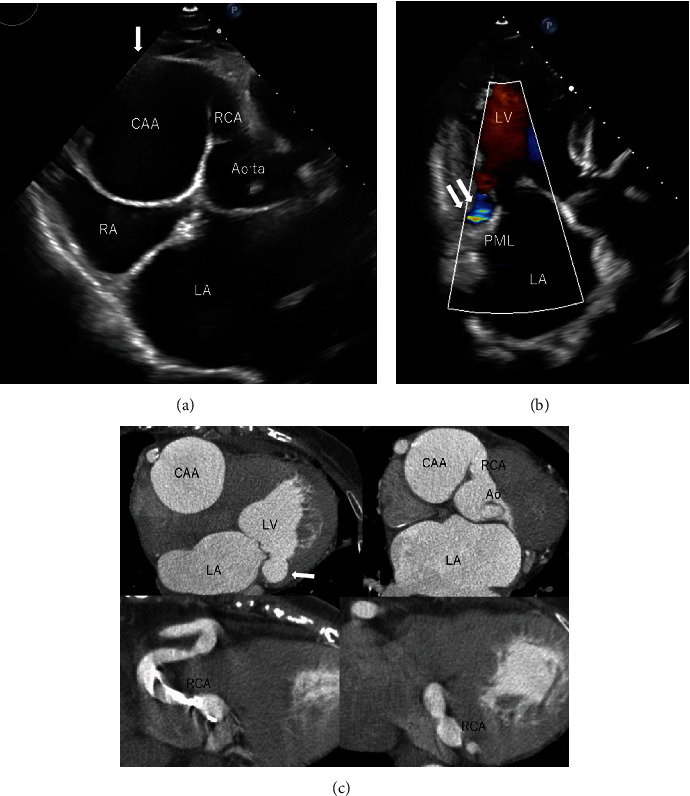
(a, b) Preoperative ultrasound echocardiography. CAA (↓) CAF (↓↓). (c) MDCT of coronary artery. CAF (↓). CAA: coronary artery aneurysm; RCA: right coronary artery; RA: right atrium; LA: left atrium; LV: left ventricle; PML: posterior mitral reflet; Ao: ascending aorta.

A 79-year-old woman, who had been diagnosed with right coronary artery- (RCA-) LV fistula by coronary angiography 20 years ago, was transferred to our hospital for evaluation of an RCA aneurysm revealed by ultrasound cardiography (Figure 1(a)). The fistula flowed into the LV underneath the posterior leaflet of the mitral valve (Figure 1(b)). Although she did not have any symptom, she also had chronic atrial fibrillation. Multidetector computed tomography (MDCT) revealed a giant RCA aneurysm and an abnormal connection between the terminal RCA and LV (Figures 1(c) and 1(d)). Although she was asymptomatic, surgical treatment was recommended because the aneurysm was large; its diameter was 50.8 mm.

## 3. Operative Procedure

**Figure 2:**
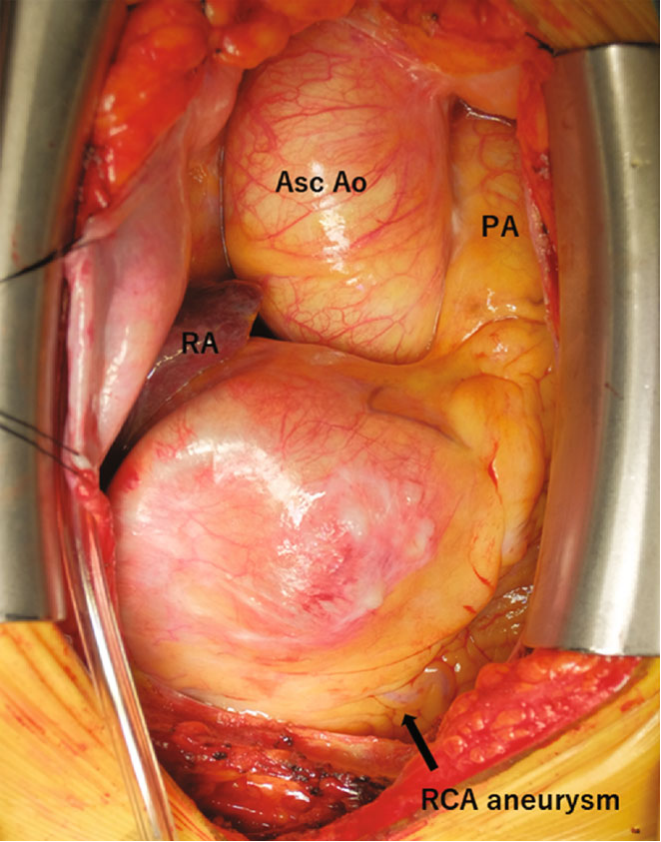
Intraoperative view. asc Ao: ascending aorta; PA: pulmonary artery.

The operation was performed via a median sternotomy. A large RCA aneurysm in the right atrium was observed after pericardiotomy (Figure 2). At the beginning of the surgery, the RCA juts distal to the CAA and encircled with vascular tape. By monitoring transesophageal echocardiography, the inferior wall motion decreased after occlusion of the RCA. Once a graftable terminal branch of the posterior descending artery (PDA) was detected, we decided to create a coronary bypass.

Surgical repair was performed under cardiac arrest with cardiopulmonary bypass. Cardiac arrest was achieved by routine antegrade cardioplegia infusion and additional antegrade infusion with RCA occlusion.

The Cryo-Maze procedure was performed via a left and right atriotomy. Although an ostium of the RCA-LV fistula was detected behind the posterior mitral leaflet, closure of the fistula seemed impossible or would be incomplete because of infraventricular structures such as the chordae tendineae.

**Figure 3:**
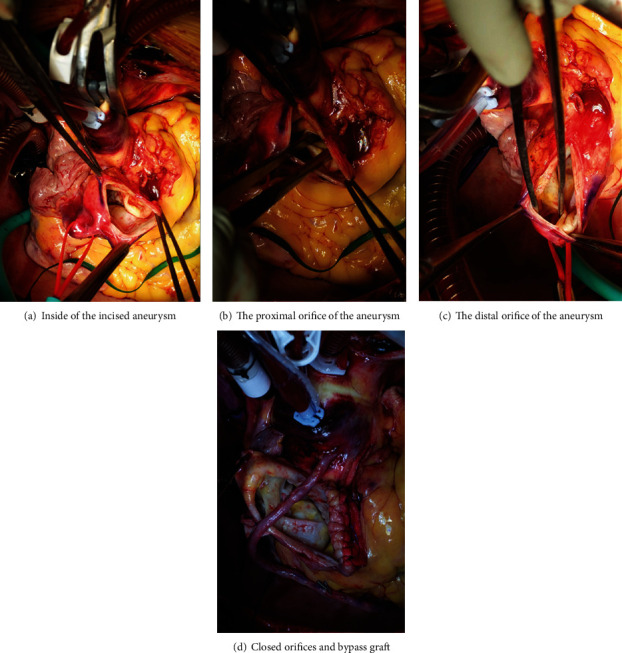
(a–d) Intraoperative images during the procedure.

The CAA was incised, and the proximal ostium was closed directly. A coronary artery bypass to the PDA was created using the saphenous vein. (Figures 3(a)–3(d)) The proximal end of the grafted PDA was ligated to avoid stealing to the left ventricle.

The distal end of the RCA aneurysm was ligated from the outside, and direct closure was added from the inside.

**Figure 4:**
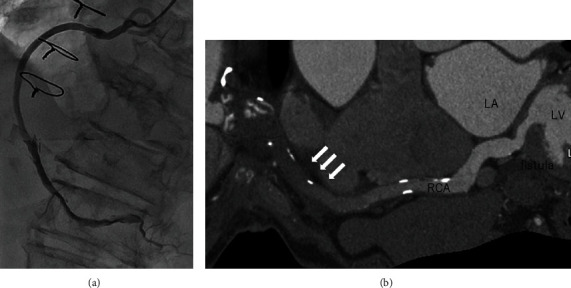
(a, b) Postoperative coronary angiography (a) and MDCT (b).

Cardiopulmonary bypass support was easily weaned, and the postoperative course was uneventful except for atrial fibrillation was sustained. Postoperative coronary angiography revealed a patent bypass graft without flow into the LV (Figure 4(a)). Postoperative coronary MDCT revealed a partially thrombosed residual RCA connected to the fistula (Figure 4(b)).

## 4. Comment

The main cause of CAA is atherosclerosis, followed by congenital inflammation, iatrogenic injury, and infection [5]. Most previous reports of CAAs with CAFs report congenital malformation as the causative factor. Although there is no consensus about the surgical indication for CAA, the aim of this treatment is prophylaxis against thromboembolism and rupture.

As for CAFs, 52% of them originate from the RCA, with the right heart being the most common draining site [6]. CAF to the LV, as in this case, is extremely rare and accounts for 4% of CAFs [7]. Volume overloading due to shunt flow, aneurysmal dilatation of the coronary artery, ischemic symptoms due to coronary steal, and so on are considered indications for treatment [4].

Moreover, the cause of our patient's CAA did not seem to be congenital as no aneurysm was detected during previous examinations. The RCA was exposed to antegrade hyperblood flow and direct retrograde systolic blood pressure via CAF, which might induce aneurysmal dilatation of the RCA and diffuse RCA ectasia. The influence of atherosclerotic degeneration could not be ruled out given her age; however, it was not detected in the histopathological examination of the resected aneurysm.

As for her atrial fibrillation, its cause might be CAA compression of the atrium. However, it might be the result of left atrial dilatation caused by similar hemodynamics to aortic regurgitation. The latter more likely to be the cause of her atrial fibrillation because her left atrium diameter was 63.0 mm in diameter preoperatively and her atrial fibrillation persisted after resection of the CAA.

Ideally, surgical correction of this anomaly should include resection of the aneurysm, reconstruction of the coronary circulation, and closure of the fistula [8]. As the contraction of the inferior wall decreased after RCA obstruction and a reasonably sized PDA was detected, a coronary artery bypass was created. If no graft vessel can be detected, systolic retrograde blood flow via the fistula may be the only remaining blood supply to the RCA. Furthermore, antegrade cardioplegia infusion with RCA obstruction was added in order to induce cardiac arrest for fear of inadequate distribution due to steal to emptying the LV via the fistula.

Since it was difficult to approach the fistula and the blood supply to the atrioventricular node artery might depend on direct blood flow from the LV in our case, closure of the fistula was finally abandoned. We investigated the prognosis of residual RCA with fistula; however, postoperative coronary MDCT revealed partially thrombosed residual RCA with a fistula. As the residual RCA close to the fistula is exposed to systemic pressure from the LV, careful long-term follow-up is essential for detection of residual fistula and RCA ectasia.
